# Supplementary material for: Unveiling the Bioactive Potential of the Invasive Jellyfish Phyllorhiza punctata Through Integrative Transcriptomic and Proteomic Analyses
Source: Biomolecules. 2025 Aug 4;15(8):1121. doi: 10.3390/biom15081121 (PMC12383608; doi:10.3390/biom15081121)
Supplement: Supplementary file 1 [file biomolecules-15-01121-s001.zip › Suplementary Material/FileS3/PEPFOLD4/XP_065067076_15100/clusters.html]

 PEP-FOLD clusters


Download archive of all models  here   

|  |  |  |
| --- | --- | --- |
| # | file | sOPEP |
| # cluster 1 - Size: 12 |  |  |
|  | XP\_065067076\_15100-model1 | -20.5311 |
|  | XP\_065067076\_15100-model1.1 | -20.2768 |
|  | XP\_065067076\_15100-model1.2 | -20.2764 |
|  | XP\_065067076\_15100-model1.3 | -20.2681 |
|  | XP\_065067076\_15100-model1.4 | -20.2647 |
|  | XP\_065067076\_15100-model1.5 | -20.2299 |
|  | XP\_065067076\_15100-model1.6 | -20.144 |
|  | XP\_065067076\_15100-model1.7 | -20.0178 |
|  | XP\_065067076\_15100-model1.8 | -20.0073 |
|  | XP\_065067076\_15100-model1.9 | -20.0073 |
|  | XP\_065067076\_15100-model1.10 | -19.694 |
|  | XP\_065067076\_15100-model1.11 | -19.5349 |
| # cluster 2 - Size: 3 |  |  |
|  | XP\_065067076\_15100-model2 | -20.5189 |
|  | XP\_065067076\_15100-model2.1 | -20.2273 |
|  | XP\_065067076\_15100-model2.2 | -20.2273 |
| # cluster 3 - Size: 13 |  |  |
|  | XP\_065067076\_15100-model3 | -16.6901 |
|  | XP\_065067076\_15100-model3.1 | -16.5493 |
|  | XP\_065067076\_15100-model3.2 | -16.4685 |
|  | XP\_065067076\_15100-model3.3 | -16.4157 |
|  | XP\_065067076\_15100-model3.4 | -16.3811 |
|  | XP\_065067076\_15100-model3.5 | -16.3273 |
|  | XP\_065067076\_15100-model3.6 | -16.3067 |
|  | XP\_065067076\_15100-model3.7 | -16.2608 |
|  | XP\_065067076\_15100-model3.8 | -16.1786 |
|  | XP\_065067076\_15100-model3.9 | -16.157 |
|  | XP\_065067076\_15100-model3.10 | -16.1253 |
|  | XP\_065067076\_15100-model3.11 | -16.1085 |
|  | XP\_065067076\_15100-model3.12 | -16.0733 |
| # cluster 4 - Size: 7 |  |  |
|  | XP\_065067076\_15100-model4 | -16.6668 |
|  | XP\_065067076\_15100-model4.1 | -16.5021 |
|  | XP\_065067076\_15100-model4.2 | -16.4733 |
|  | XP\_065067076\_15100-model4.3 | -16.4188 |
|  | XP\_065067076\_15100-model4.4 | -16.3807 |
|  | XP\_065067076\_15100-model4.5 | -16.3512 |
|  | XP\_065067076\_15100-model4.6 | -16.2223 |
| # cluster 5 - Size: 34 |  |  |
|  | XP\_065067076\_15100-model5 | -16.6126 |
|  | XP\_065067076\_15100-model5.1 | -16.5846 |
|  | XP\_065067076\_15100-model5.2 | -16.5433 |
|  | XP\_065067076\_15100-model5.3 | -16.5394 |
|  | XP\_065067076\_15100-model5.4 | -16.5307 |
|  | XP\_065067076\_15100-model5.5 | -16.5218 |
|  | XP\_065067076\_15100-model5.6 | -16.5218 |
|  | XP\_065067076\_15100-model5.7 | -16.5079 |
|  | XP\_065067076\_15100-model5.8 | -16.4921 |
|  | XP\_065067076\_15100-model5.9 | -16.4612 |
|  | XP\_065067076\_15100-model5.10 | -16.4265 |
|  | XP\_065067076\_15100-model5.11 | -16.4023 |
|  | XP\_065067076\_15100-model5.12 | -16.3827 |
|  | XP\_065067076\_15100-model5.13 | -16.3653 |
|  | XP\_065067076\_15100-model5.14 | -16.3549 |
|  | XP\_065067076\_15100-model5.15 | -16.3438 |
|  | XP\_065067076\_15100-model5.16 | -16.3382 |
|  | XP\_065067076\_15100-model5.17 | -16.3374 |
|  | XP\_065067076\_15100-model5.18 | -16.3302 |
|  | XP\_065067076\_15100-model5.19 | -16.3281 |
|  | XP\_065067076\_15100-model5.20 | -16.324 |
|  | XP\_065067076\_15100-model5.21 | -16.3121 |
|  | XP\_065067076\_15100-model5.22 | -16.3023 |
|  | XP\_065067076\_15100-model5.23 | -16.2924 |
|  | XP\_065067076\_15100-model5.24 | -16.2618 |
|  | XP\_065067076\_15100-model5.25 | -16.2499 |
|  | XP\_065067076\_15100-model5.26 | -16.2396 |
|  | XP\_065067076\_15100-model5.27 | -16.2382 |
|  | XP\_065067076\_15100-model5.28 | -16.2382 |
|  | XP\_065067076\_15100-model5.29 | -16.2299 |
|  | XP\_065067076\_15100-model5.30 | -16.2083 |
|  | XP\_065067076\_15100-model5.31 | -16.1726 |
|  | XP\_065067076\_15100-model5.32 | -16.1647 |
|  | XP\_065067076\_15100-model5.33 | -16.0582 |
| # cluster 6 - Size: 27 |  |  |
|  | XP\_065067076\_15100-model6 | -16.607 |
|  | XP\_065067076\_15100-model6.1 | -16.5019 |
|  | XP\_065067076\_15100-model6.2 | -16.4403 |
|  | XP\_065067076\_15100-model6.3 | -16.4207 |
|  | XP\_065067076\_15100-model6.4 | -16.3922 |
|  | XP\_065067076\_15100-model6.5 | -16.3802 |
|  | XP\_065067076\_15100-model6.6 | -16.3602 |
|  | XP\_065067076\_15100-model6.7 | -16.3593 |
|  | XP\_065067076\_15100-model6.8 | -16.3349 |
|  | XP\_065067076\_15100-model6.9 | -16.318 |
|  | XP\_065067076\_15100-model6.10 | -16.3173 |
|  | XP\_065067076\_15100-model6.11 | -16.3107 |
|  | XP\_065067076\_15100-model6.12 | -16.2983 |
|  | XP\_065067076\_15100-model6.13 | -16.2836 |
|  | XP\_065067076\_15100-model6.14 | -16.2816 |
|  | XP\_065067076\_15100-model6.15 | -16.2782 |
|  | XP\_065067076\_15100-model6.16 | -16.2776 |
|  | XP\_065067076\_15100-model6.17 | -16.2685 |
|  | XP\_065067076\_15100-model6.18 | -16.2576 |
|  | XP\_065067076\_15100-model6.19 | -16.2513 |
|  | XP\_065067076\_15100-model6.20 | -16.2366 |
|  | XP\_065067076\_15100-model6.21 | -16.2344 |
|  | XP\_065067076\_15100-model6.22 | -16.1828 |
|  | XP\_065067076\_15100-model6.23 | -16.172 |
|  | XP\_065067076\_15100-model6.24 | -16.1635 |
|  | XP\_065067076\_15100-model6.25 | -16.1329 |
|  | XP\_065067076\_15100-model6.26 | -15.9935 |
| # cluster 7 - Size: 2 |  |  |
|  | XP\_065067076\_15100-model7 | -16.4017 |
|  | XP\_065067076\_15100-model7.1 | -16.3783 |
| # cluster 8 - Size: 1 |  |  |
|  | XP\_065067076\_15100-model8 | -16.4002 |
| # cluster 9 - Size: 1 |  |  |
|  | XP\_065067076\_15100-model9 | -16.354 |
|  |  |  |
